# Ketorolac-induced peptic ulcer bleeding after cholecystectomy in a “healed” ulcer patient: A CARE case report

**DOI:** 10.1097/MD.0000000000048355

**Published:** 2026-04-17

**Authors:** Lifen Jin, Liping Yang, Shunhua Qiu

**Affiliations:** a Department of Pharmacy, Zigong Third People’s Hospital, Zigong, Sichuan, China; b Department of Clinical Laboratory, Zigong Third People’s Hospital, Zigong, Sichuan, China.

**Keywords:** adverse drug reaction, gastrointestinal bleeding, ketorolac tromethamine, peptic ulcer

## Abstract

**Rationale:**

Ketorolac tromethamine injection-induced peptic ulcer with bleeding remains rarely reported in clinical case studies. Controversy persists, particularly regarding its analgesic use in patients with a history of gastric ulcer, even after clinical cure. Herein, this article presents a case of peptic ulcer with bleeding after supra-therapeutic dosage of ketorolac tromethamine injection for post‑cholecystectomy analgesia in a patient with a clinically “healed” gastric ulcer, emphasizing the need for cautious and rational prescribing.

**Patient concerns:**

A 70-year-old male patient with a history of peptic ulcer and gallstones presented with recurrent right upper quadrant pain for over 2 years. Previous treatment with oral Xiaoyan Lidan Tablets (an anti-inflammatory and choleretic agent) did not prevent symptom recurrence.

**Diagnoses:**

Peptic ulcer with bleeding, Gallstones with chronic cholecystitis, Hypertension (Grade 2).

**Interventions:**

The patient underwent a laparoscopic cholecystectomy under general anesthesia. For postoperative analgesia, intramuscular ketorolac tromethamine injection was administered at 30 mg twice daily, which exceeded the recommended dosage range. Appropriate symptomatic therapies, including anti‑infection, hepatoprotection, hemostasis, and fluid replacement therapy, were administered as clinically indicated. On the fourth postoperative day, the patient developed gastrointestinal bleeding with a hemoglobin level of 62 g/L. Based on the medication history, the gastrointestinal bleeding was considered associated with the application of ketorolac tromethamine injection. The drug was discontinued for observation, and the patient was started on pantoprazole for acid suppression and gastric protection, along with carbazochrome sodium sulfonate for hemostasis. As the gastrointestinal bleeding symptoms did not improve, the regimen was adjusted to esomeprazole for acid suppression and gastric protection, supplemented with hemostatic therapy using somatostatin and hemocoagulase. Following this adjustment, the patient gastrointestinal bleeding symptoms gradually improved.

**Outcomes:**

Gastrointestinal bleeding was controlled, and the patient was discharged following clinical improvement.

**Lessons:**

This case supports a potential causal relationship between the use of ketorolac tromethamine injection and the occurrence of peptic ulcer with bleeding, especially in patients with clinically cured gastric ulcer: a risk often overlooked in drug selection. Particular emphasis should be placed on individualized medication, risk assessment, and multidisciplinary collaboration to enhance patient safety and treatment efficacy.

## 1. Introduction

Ketorolac tromethamine injection represents a clinically commonly used non-steroidal anti-inflammatory drug (NSAID). Its core mechanism of action involves the nonselective inhibition of cyclooxygenase-1 (COX-1) and cyclooxygenase-2 (COX-2) activity, exerting potent analgesic and anti-inflammatory effects by reducing prostaglandin synthesis. This drug exhibits particularly prominent inhibitory activity against COX-1, with a half-maximal inhibitory concentration (IC_50_) of approximately 0.02 μM, ranking it among the NSAIDs with stronger COX-1 inhibitory potency.^[1]^ It is now extensively used clinically for analgesia, anti-inflammation, and perioperative pain management.^[2,3]^ With potent analgesic activity, it is especially effective in postoperative analgesia and renal colic.^[4]^ However, as its clinical use has increased, its potential adverse reactions, particularly those affecting the gastrointestinal tract, have garnered growing attention. Notably, a stratified analysis was conducted by carrying out a post-marketing non-randomized follow-up study of ketorolac tromethamine, involving approximately 10,000 perioperative patients. Results showed that among patients aged ≥65 years with a history of healed ulcers, the recurrence risk of severe gastrointestinal bleeding was 2.8%. In contrast, the incidence risk of this severe adverse event reached 7.8% in patients aged <65 years.^[5]^ This article analyzed and reported a case of peptic ulcer bleeding induced by ketorolac in a patient with a “healed” ulcer history, aiming to provide a reference for safer clinical medication practices.

## 2. Case report

A 70-year-old male patient, weighing 70 kg and measuring 150 cm in height, was admitted at 09:22 on March 11, 2021, due to “recurrent right upper abdominal pain for over 2 years.” The pain, which began over 2 years ago, was intermittent, dull, and distending in nature, occurred in relation to fatty meal intake, and radiated to the back. It was accompanied by nausea and reduced food intake, with no vomiting, fever, chills, acid reflux, belching, diarrhea, constipation, or other discomforts. The patient had a history of a gastric ulcer, which improved after anti-ulcer medication (details unspecified). He also had a history of gallstones and had previously taken anti-inflammatory and cholagogic tablets, but his symptoms recurred. He was admitted for further surgical treatment, with a preoperative outpatient diagnosis of “cholecystolithiasis with chronic cholecystitis.” The patient had a history of hypertension for over 2 years, managed with amlodipine besylate 2.5 mg orally once daily. Besides, he had no history of diabetes, coronary heart disease, or food and drug allergies.

**Physical examination on admission:** Temperature 36.2°C, pulse 67 beats/minute, respiratory rate 20 breaths/minute, and blood pressure 145/82 mm Hg. The patient was conscious and cooperative. No enlargement of the bilateral thyroid gland was noted, and cardiopulmonary examination revealed no abnormalities. The abdomen was flat and soft, with no tenderness or palpable masses. No costovertebral angle tenderness or shifting dullness was detected. Bowel sounds were normal. All joints exhibited a full range of motion, and no lower extremity edema was observed. Admission diagnoses: Cholecystolithiasis with chronic cholecystitis, Hypertension, grade 2.

### 2.1. Post-admission investigations

**Laboratory tests:** C-reactive protein: 25.14 mg/L (reference range: 0–10 mg/L); alanine aminotransferase: 94 U/L (9–50 U/L); aspartate aminotransferase: 82 U/L (0–40 U/L); direct bilirubin: 8.1 μmol/L (0–6 μmol/L); total bile acid: 10.8 μmol/L (0–10 μmol/L); hemoglobin: 124 g/L (130–175 g/L). The fecal occult blood test was negative. Ultrasound: liver cyst, slightly thickened gallbladder wall, suspected common bile duct stone with intra- and extrahepatic biliary dilation, right renal cyst, benign prostatic hyperplasia with calcification. Computed tomography: common bile duct stone. Gastroscopy: superficial gastritis, raised-depressed lesion in the gastric antrum, no active ulcer was seen (Figure 1.1~Figure 1.3). Further biopsy of the gastric antrum lesion revealed mild chronic inflammation with no evidence of malignancy (Fig. 2). No other significant abnormalities were identified in additional investigations.

**Figure 1.**
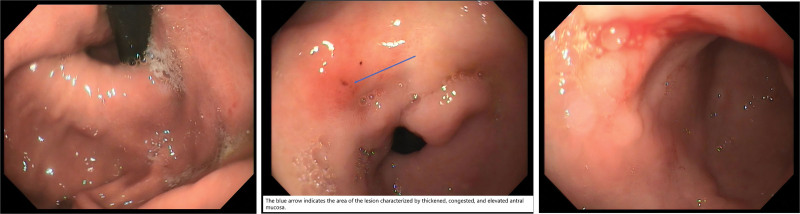
Preoperative gastroscopy (fundus of stomach). Preoperative gastroscopy (gastric antrum). The blue arrow indicates the area of the lesion characterized by thickened, congested, and elevated antral mucosa. Preoperative gastroscopy (body of stomach).

**Figure 2.**
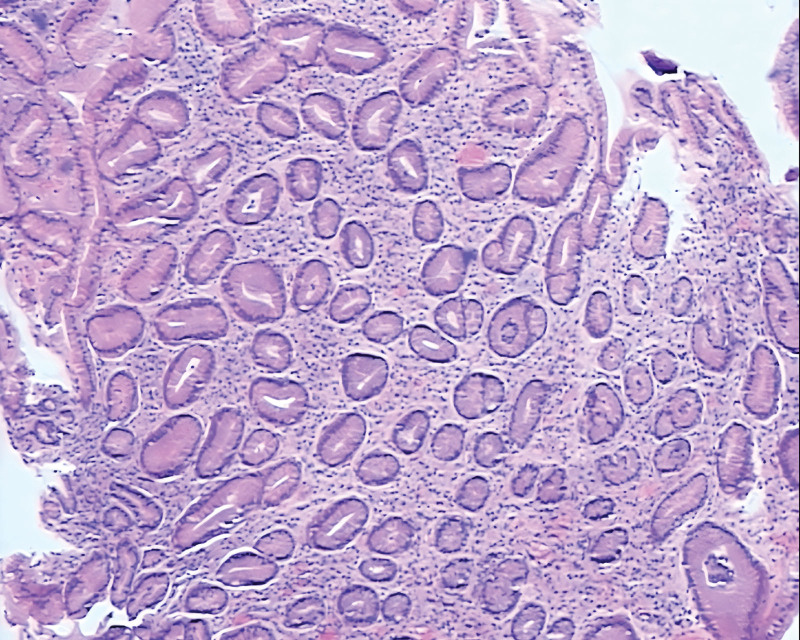
Pathological biopsy (Hematoxylin and Eosin stain [H&E]).

**Anesthesia Evaluation:** The patient blood pressure was 136/82 mm Hg, and he had a history of gastric ulcer with no active manifestations. The assessed anesthesia risk was low, classified as American Society of Anesthesiologists physical status class II. Comprehensive evaluation confirmed surgical indications with no remarkable contraindications. Repeat fecal occult blood test was negative. Subsequently, the patient underwent an uneventful laparoscopic cholecystectomy under general anesthesia on March 16, 2021.

### 2.2. Postoperative management

The patient complained of incisional and upper abdominal pain. Ketorolac tromethamine injection 30 mg was administered intramuscularly twice daily for analgesia, along with routine symptomatic treatments including anti-infection therapy, liver protection, hemostasis, and fluid replacement.

At 06:40 on postoperative day 4 (March 20), the patient reported passing tarry stool 3 times, with a total volume of approximately 500 grams. The fecal occult blood test was positive, and the hemoglobin was 62 g/L (representing a drop of 62 g/L from admission, exceeding 20 g/L). Upper gastrointestinal bleeding associated with nonsteroidal analgesic use was suspected. Ketorolac was immediately discontinued, and treatment was initiated with pantoprazole 40 mg once daily via intravenous drip for acid suppression, along with carbazochrome sodium sulfonate for hemostasis. Additionally, 2 units of suspended red blood cells were transfused.

On postoperative day 5 (March 21), the patient continued to pass black stools. Emergency blood tests revealed: C-reactive protein: 70.52 mg/L; neutrophils: 70.6% (normal range: 40%–70%); red-cell count: 2.10 × 10^12^/L (4.3–5.8 × 10^12^/L); hemoglobin: 63 g/L; hematocrit: 19.30%. Treatment was continued with pantoprazole 40 mg intravenously twice daily for acid suppression and hemostasis, and an additional 2 units of suspended red blood cells were transfused.

At 04:30 on postoperative day 6 (March 22), the patient passed approximately 100 ml of fresh red bloody stool once. A gastroenterology consultation was requested. The consultants recommended the following diagnoses: peptic ulcer with bleeding and moderate hemorrhagic anemia. For consultation opinion, fasting, gastrointestinal decompression, and intensive acid suppression and hemostasis were recommended. Norepinephrine combined with prothrombin complex concentrate may be administered via gastric tube if required. Treatment was switched to esomeprazole 8 mg/h continuous intravenous infusion, combined with comprehensive hemostatic therapy including carbazochrome sodium sulfonate, somatostatin, and hemocoagulase.

**Figure 3.**
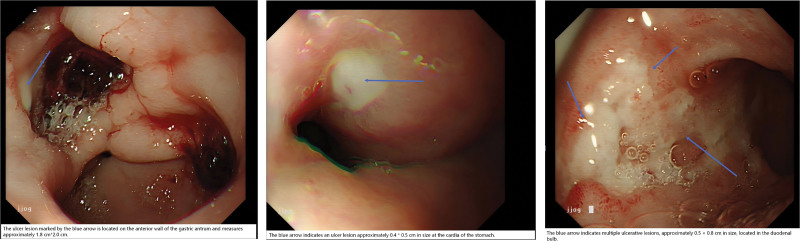
Endoscopy after medication (gastric antrum). The ulcer lesion markedby the blue arrow is located on the anterior wall of the gastric antrum and measures approximately 1.8 cm × 2.0 cm. Endoscopy after medication (cardia of stomach). The blue arrow indicates an ulcer lesion approximately 0.4 × 0.5 cm in size at the cardia of the stomach. Endoscopy after medication (duodenal bulb). The blue arrow indicates multiple ulcerative lessions approximately 0.5 × 0.8 cm in size, located in the duodenal bulb.

On postoperative day 7 (March 23), an emergency gastroscopy revealed multiple ulcers in the cardia, gastric antrum, and duodenal bulb. The gastric antral ulcer was particularly large and deep, accompanied by slight oozing of blood and attached blood clots (Figure 3). The ultimate diagnosis was a peptic ulcer with bleeding.

Upon implementing the recommended treatments, the patient had no further hematochezia by postoperative day 11, and the repeat hemoglobin was 72 g/L. The patient general condition has gradually improved and is currently stable. (Table 1)

**Table 1:** Case diagnosis timeline.

Date	Medical course
On March 11, 2021	The patient was admitted. Comprehensive medical history collection, physical examination, laboratory tests, imaging studies, and gastroscopy were performed.
On March 16, 2021	Laparoscopic cholecystectomy was performed under general anesthesia. On the day of surgery, 30 mg of ketoprofen tromethamine injection was administered intramuscularly for pain control at 18:04.
On March 17, 2021 (postoperative d 1)	At 00:05, 30 mg of ketorolac tromethamine injection was administered intramuscularly. At 15:30, 30 mg of ketorolac tromethamine injection was administered intramuscularly.
On March 18, 2021 (postoperative d 2)	At 12:52, 30 mg of ketorolac tromethamine injection was administered intramuscularly. At 19:15, 30 mg of ketorolac tromethamine injection was administered intramuscularly.
On March 19, 2021 (postoperative d 3)	At 00:40, 30 mg of ketorolac tromethamine injection was administered intramuscularly. At 19:47, the final dose of ketorolac tromethamine injection 30 mg was administered intramuscularly.
On March 20, 2021 (postoperative d 4)	At 06:40, the patient passed tarry stool 3 times, with a total volume of approximately 500 g. A fecal occult blood test returned positive, and the hemoglobin level was 62 g/L. Considering upper gastrointestinal bleeding associated with nonsteroidal analgesic use, ketorolac was immediately discontinued. Treatment was initiated with pantoprazole for acid suppression and carbazochrome sodium sulfonate for hemostasis, along with a transfusion of 2 units of suspended red blood cells.
On March 21, 2021 (postoperative d 5)	The patient continued to pass black stools. An urgent complete blood count was performed. Acid suppression and hemostatic therapies were continued, and 2 units of suspended red blood cells were transfused.
On March 22, 2021 (postoperative d 6)	Gastrointestinal bleeding worsened. The treatment was switched to continuous infusion of esomeprazole at 8 mg/h, combined with comprehensive hemostatic measures including carbazochrome sodium sulfonate, somatostatin, and hemocoagulase.
On March 23, 2021 (postoperative d 7)	Emergency gastroscopy was performed, and the diagnosis was confirmed as a peptic ulcer with bleeding.
On March 27, 2021 (postoperative d 11)	The patient condition improved.

## 3. Discussion

### 3.1. Evaluation of adverse drug reaction (ADR) association

An association evaluation was conducted for ketorolac tromethamine injection-induced peptic ulcer with bleeding: temporal relationship. The patient received ketorolac tromethamine injection for analgesia postcholecystectomy, and the ADR (gastrointestinal bleeding) occurred 4 days following drug administration. This indicated a plausible temporal relationship between the medication and the adverse event; known reaction type, the drug prescribing information explicitly lists post-marketing adverse reaction reports including peptic ulcer, gastrointestinal bleeding, melena, and gastrointestinal perforation, confirming it as a known type of adverse reaction; response to dechallenge, following discontinuation of ketorolac and institution of symptomatic therapy (acid suppression, gastric protection, and hemostasis), the patient ADR symptoms improved markedly; no rechallenge, The drug was not readministered; stress-related mucosal lesions typically occur more than 48 hours after major trauma/sepsis and are more commonly observed in ICU patients. The patient in this case exhibited no independent risk factors. According to the clinical risk scoring system for gastrointestinal bleeding due to stress-related mucosal lesions, the patient score was calculated as follows: age >60 years (2 points), male gender (2 points), history of hypertension (3 points), totaling 7 points, which classified him as low-risk. Therefore, surgery-induced stress-related mucosal lesions could be excluded.^[6]^ Based on the above analysis and according to the association evaluation criteria in the *Provisions for Adverse Drug Reaction Reporting and Monitoring from the National Center for ADR Monitoring*,^[7]^ the association between this event of peptic ulcer with bleeding and ketorolac tromethamine injection was evaluated as “Probable.” According to the Naranjo ADR Probability Scale,^[8]^ the causality assessment yielded a total score of 7, indicating a “probable” relationship between the adverse event and the drug. The detailed scoring was calculated as follows: the onset of peptic ulcer with bleeding occurred following administration of ketorolac tromethamine injection (2 points); the condition improved following discontinuation of the drug and symptomatic treatment (1 point); the prescribing information for ketorolac tromethamine injection explicitly lists peptic ulcer and gastrointestinal bleeding as known adverse reactions (1 point); no other independent causes could fully explain the occurrence of peptic ulcer with bleeding in this patient (2 points); initial gastroscopy on admission revealed no ulceration; hemoglobin was 124 g/L, and 2 fecal occult blood tests were negative. These findings served as objective evidence supporting the association (1 point).

### 3.2. Analysis of prevention of clinical medication errors

The dosage of ketorolac tromethamine injection should be individualized based on patient age, weight, and route of administration. The prescribing information clearly recommends a single intramuscular dose of 30 mg per day for patients aged 65 and older; for multiple daily doses, 15 mg every 6 hours is recommended, with a maximum daily dose not exceeding 60 mg and treatment duration not surpassing 5 days. For instance, Strom BL et al evaluated the risk of gastrointestinal and surgical‑site bleeding associated with ketorolac tromethamine injection. They observed that higher doses administered for >5 days further increased the risk of bleeding and adverse events in elderly patients. In this case, the patient was a 70‑year‑old male who received ketorolac tromethamine 30 mg intramuscularly twice daily after cholecystectomy. The excessive single dose likely contributed to the development of a peptic ulcer with bleeding. Therefore, clinicians must strictly adhere to the recommended dosage in the prescribing information, taking into account patient age and administration route, so as to avoid adverse reactions that may arise from overdosing. Additionally, electronic prescribing systems should implement mandatory alerts to block inappropriate orders: for patients aged ≥65 years, single intramuscular doses exceeding 30 mg (once‑daily) or 15 mg (multiple‑daily) should be prevented. Pharmacists should reinforce verification of patient age, single‑dose amount, and maximum daily dose when reviewing ketorolac tromethamine prescriptions to prevent overdosing.

### 3.3. Analysis of clinical medication safety risk assessment and rational use

Ketorolac tromethamine injection is contraindicated in patients with active peptic ulcer, recent gastrointestinal bleeding or perforation, or a history of peptic ulcer or gastrointestinal bleeding. The correlation between intramuscular ketorolac tromethamine and gastrointestinal complications in elderly patients requires that ketorolac should be avoided in patients with a history of gastric ulcer. Prophylactic misoprostol is advised for high-risk patients administered ketorolac.^[9]^ As recommended by the *Guidelines for the Prevention and Treatment of NSAID‑Associated Gastrointestinal Ulcers and Ulcer Complications*, all patients scheduled for elective surgery undergo active assessment of gastrointestinal bleeding risk prior to NSAID use. High‑risk patients should avoid non‑selective NSAIDs, and selective COX‑2 inhibitors may be considered, optionally combined with misoprostol or a proton‑pump inhibitor to reduce the risk of gastrointestinal bleeding.^[10]^ Another study reported that the cumulative annual incidence of gastrointestinal bleeding was 12% in triple‑risk NSAID users, as opposed to 1.5% in those without risk factors.^[11]^ In this case, the patient was at high-risk of gastrointestinal bleeding due to age >65 years, history of hypertension, and a documented gastric ulcer 2 years earlier. Gastroscopy on admission revealed superficial gastritis and a depressed elevated lesion in the gastric antrum, with no active ulcer, indicating a healed gastric ulcer. This finding may lead clinicians to misinterpret “no active ulcer” as “no risk.” However, the patient developed a peptic ulcer with bleeding on day 4 following initiation of ketorolac tromethamine injection, leading to clinical deterioration. This further substantiates that patients with clinically healed peptic ulcers or prior gastrointestinal bleeding remain at significantly high-risk of ulceration or bleeding when exposed to ketorolac. A retrospective comparative study found that intravenous acetaminophen presented analgesic efficacy similar to intravenous ketorolac, particularly in patients with relative or absolute contraindications to ketorolac, establishing it as a more beneficial alternative.^[12]^ A literature review indicated that intravenous acetaminophen exhibited fewer adverse effects and superior safety compared with ketorolac at therapeutic doses.^[13]^ Therefore, for patients with a history of peptic ulcer or gastrointestinal bleeding (even if clinically healed), ketorolac tromethamine should not be recommended for perioperative analgesia. Intravenous acetaminophen may serve as a reasonable alternative.

To summarize, this case suggests a probable association between ketorolac tromethamine injection and the occurrence of peptic ulcer with bleeding. Clinicians should thoroughly assess patients’ gastrointestinal risk profiles prior to ketorolac administration, strictly adhere to the approved indications, dosage, and administration outlined in the prescribing information, and control treatment duration accordingly. Ketorolac is contraindicated in patients with established contraindications. For postoperative patients, the risks and benefits must be carefully weighed, safer alternative analgesics should be selected, or gastric mucosal protective measures (e.g., proton pump inhibitors) should be implemented to reduce the risks of mucosal injury.^[14]^ Close monitoring should be maintained throughout treatment for early detection of ADRs. Individualized medication, risk assessment, and multidisciplinary collaboration are essential to optimize patient safety and clinical outcomes.

## Author contributions

**Writing – original draft:** Lifen Jin, Shunhua Qiu.

**Writing – review & editing:** Lifen Jin, Liping Yang, Shunhua Qiu.
